# A Tunable Hyperspectral Imager for Detection and Quantification of Marine Biofouling on Coated Surfaces

**DOI:** 10.3390/s22187074

**Published:** 2022-09-19

**Authors:** Joaquim Santos, Morten Lysdahlgaard Pedersen, Burak Ulusoy, Claus Erik Weinell, Henrik Chresten Pedersen, Paul Michael Petersen, Kim Dam-Johansen, Christian Pedersen

**Affiliations:** 1 Department of Electrical and Photonics Engineering (DTU Electro), Technical University of Denmark, 4000 Roskilde, Denmark; 2 CoaST, Department of Chemical and Biochemical Engineering (DTU Chemical Engineering), Technical University of Denmark, 2800 Kongens Lyngby, Denmark; 3 Sino-Danish Center for Education and Research, Beijing 100093, China; 4 Sino-Danish College, University of Chinese Academy of Sciences, Beijing 100049, China

**Keywords:** hyperspectral imaging, biofouling, spectral library, classification, fouling control coatings, led illumination, pixelwise calibration

## Abstract

Fouling control coatings (FCCs) are used to prevent the accumulation of marine biofouling on, e.g., ship hulls, which causes increased fuel consumption and the global spread of non-indigenous species. The standards for performance evaluations of FCCs rely on visual inspections, which induce a degree of subjectivity. The use of RGB images for objective evaluations has already received interest from several authors, but the limited acquired information restricts detailed analyses class-wise. This study demonstrates that hyperspectral imaging (HSI) expands the specificity of biofouling assessments of FCCs by capturing distinguishing spectral features. We developed a staring-type hyperspectral imager using a liquid crystal tunable filter as the wavelength selective element. A novel light-emitting diode illumination system with high and uniform irradiance was designed to compensate for the low-filter transmittance. A spectral library was created from reflectance-calibrated optical signatures of representative biofouling species and coated panels. We trained a neural network on the annotated library to assign a class to each pixel. The model was evaluated on an artificially generated target, and global accuracy of 95% was estimated. The classifier was tested on coated panels (exposed at the CoaST Maritime Test Centre) with visible intergrown biofouling. The segmentation results were used to determine the coverage percentage per class. Although a detailed taxonomic description might be complex due to spectral similarities among groups, these results demonstrate the feasibility of HSI for repeatable and quantifiable biofouling detection on coated surfaces.

## 1. Introduction

One of the greatest threats to marine biodiversity on a worldwide scale is the global spread of non-indigenous species (NIS) [1,2,3]. NIS are species of organisms that have been introduced in regions outside of their natural range and natural dispersal potential [2,4], mainly through ballast water or as hull biofouling [1,5]. Marine biofouling, or simply biofouling, is defined in [6] as “the undesirable accumulation of microorganisms, plants, and animals on artificial surfaces immersed in sea water”. Aside from globally spreading NIS, biofouling on ship hulls also induces other negative effects, such as increased frictional resistance, loss of maneuverability, and decreased fuel efficiency [6,7,8]. To help reduce these problems, the shipping and other offshore industries apply fouling control coatings (FCCs) [9] as top coats on various submerged artificial surfaces. The term FCC covers different types of coatings utilizing different mechanisms to prevent biofouling on, e.g., ship hulls. Two common types are self-polishing and fouling-release coatings. Self-polishing coatings contain biocides, which are released through controlled surface polishing and inhibit or limit the settlement of biofouling [8,10]. Fouling-release coatings use low surface free energy and high elasticity, which weaken the adhesion of biofouling and promote its removal by the shear force of water [10].

The testing and evaluations of FCCs are done according to different standards, e.g., the Naval Ships’ Technical Manual (NSTM) [11], Standard Test Method for Testing Antifouling Panels in Shallow Submergence (ASTM-D3623) [12], or European Chemicals Agency (ECHA) [13]. These standards provide information about test conditions and procedures for the evaluation of FCC performances. Despite having different approaches on how to evaluate performances, a common denominator for the three standards is the manual assessment based on visual inspection of coated test panels. This means that the basis for evaluation, e.g., a biofouling pattern (NSTM), number/size, or coverage percentage (ASTM-D3623, ECHA), comes from an examination performed by an investigator, usually an expert with notable knowledge of local marine ecosystems and, therefore, is susceptible to latent subjectivity and bias [14,15,16]. This subjectivity affects repeatability, as different examiners will inevitably provide different coverage percentages even on the same sample. Furthermore, by being a manual task, it is also time-consuming and prone to errors. In this sequence, the development of new objective and repeatable methods for evaluation of the performances of FCCs is a crucial step in the roadmap to minimize the spread of NIS and the emission of greenhouse gases through increased fuel consumption [15,16].

Throughout the past decade, the use of image analysis, in conjunction with increased access to computational power and developments in supervised classification algorithms [17,18], have advanced many fields requiring object detection and identification. Among these, biofouling evaluation has benefited from the implementation of automated methodologies relying on image analysis to attain reliable and objective results. In particular, a very recent application (of deep learning to underwater images of vessels) demonstrated the feasibility and effectiveness of automated image analysis for biofouling detection [15]. However, this approach was only semi-quantitative, providing a holistic classification according to a three-level scale (no fouling; 1–15% coverage, 15–100% coverage). In the context of FCC performance assessment, having finer information on the coverage percentage of both biofouling as a whole (and with specific groups) is important to support the development stages and the direct comparison between different coatings. Attempting to increase the classification specificity, more detailed supervised models have been trained, particularly support vector machines (SVMs) [19] and pixel classification models [16]. In the former case, in situ and in vivo images were acquired and a spectral library was built with several classes (clean panel, algae, encrusting tunicates and bryozoans, cnidarian polyps, and other fouling). Overall accuracy of 58% was achieved by the model. In the latter case, biofouling was classified as microalgae, macroalgae, animals, and panel.

All of the aforementioned methods build upon RGB images and, therefore, confusion occurs between different groups with similar hues and brightness [20]. Moreover, the obtained specificity class-wise is restricted due to the limited information provided by these techniques. In this paper, we explore and demonstrate the suitability of HSI as a tool to detect and quantify marine biofouling on submerged coated surfaces. Hyperspectral cameras integrate monochromatic imaging sensors with dispersive elements to combine two-dimensional spatial information (*x*,*y*) and spectral information (λ) of a scene in a three-dimensional data structure called a hypercube. This structure consists of a stack of multiple 2D quasi-monochromatic images in which each slice is indexed to a well-defined wavelength band. Four HSI architectures are broadly acknowledged according to the acquisition scheme [21]: whisk-broom or point scan, push broom or line scanning, staring, and snapshot.

Since the interaction between light from different bands of the electromagnetic spectrum and matter depends on the properties of the object, such as composition and geometry, the scattered and fluorescent light captured within the field-of-view (FOV) carries information about the scene [21]. These spectrally-rich data are suitable for classification purposes as they can be compared at the pixel level with known optical signatures of the desired objects [22]. By increasing the number of channels from three to several tens, the amount of information at the pixel level is remarkably extended. This way, HSI can resolve narrow spectral fingerprints in the reflectance of target groups [23,24], which are otherwise missed by conventional RGB cameras [25], as, for instance, chlorophyll-a (chl-a) absorption and fluorescence [26]. These additional features can be used as bio-optical identification tools to discriminate between biofouling classes and have been shown to improve classification accuracy [20].

Despite being a well-established, mature, and widely employed technology for remote oceanographic surveying from space and airborne platforms [27,28,29], underwater deployment of hyperspectral imagers in marine media is a relatively recent endeavor. Several in situ applications with high spatial resolutions (sub-mm [30]) have been propelled by the advent of the first underwater push broom hyperspectral system developed by Ecotone AS [31]. The potential of this technology has been demonstrated for microbial biofilm quantification in intertidal sediments [30], mapping of benthic habitats, surveying of ecosystems [20,22,25], and mineral exploration [32,33].

Imaging through water is challenging due to the inherent optical properties of seawater that limit operations to the visible range of the electromagnetic spectrum [34], as strong absorption outside this window largely reduces photon budgets. For the same underlying reason, underwater HSI systems usually require active illumination from artificial light sources to provide a sufficient signal-to-noise ratio (SNR). These light sources must cover the full operational spectrum and be stable, efficient, compact, powerful, and spatially uniform, to avoid local degradation of image quality [35]. Furthermore, since absorption by water is wavelength-dependent, and adding scattering and fluorescent effects from suspended matter, the measured spectra are distorted after propagation through the medium. Hence, calibration is mandatory to eliminate these influences and retrieve an accurate estimate of the reflectance spectrum of objects [33,36].

The aim of the present work was to develop an objective, repeatable, and time-efficient system to evaluate the performances of FCCs with higher specificity class-wise when compared to existing manual and RGB-based methods. To fulfill this goal, we developed a lab-bench staring-type HSI system using a liquid crystal tunable filter (LCTF) as a spectral-resolving element, justified by the stationary nature of the application. We also opted for a LCTF due to the high imaging quality and swift tuning speeds [21]. A custom light source with a novel design was built to achieve high and uniform intensity at an underwater imaging plane and, thus, to fasten acquisitions. We built a spectral library of representative biofouling species at the CoaST Maritime Test Centre (CMTC). In a two-step processing, we applied the principal component analysis (PCA) to compare the collected signatures and subsequently used them as the training set for a supervised machine learning model for automatic classification. The accuracy of the resulting classifier was evaluated on a model target with known ground truth. The classification performance was also assessed in real panels previously exposed to seawater for several months to map the coverage percentage of different biofouling groups. The developed method captured characteristic spectral fingerprints of biofouling that were crucial to increase specificity class-wise and to determine the respective coverage percentages present on coated surfaces.

This paper is structured as follows: Section 2 presents the developed HSI system, which includes a novel uniform and intense LED light source, the sample collection and measurement procedures, and the subsequent data analysis workflow to calibrate, classify, and quantify biofouling. Section 3 reports on the results, and Section 4 closes with the discussion and concluding remarks.

## 2. Materials and Methods

### 2.1. Test Site and Materials

The biofouling used in this work was obtained from the CMTC. CMTC is a floating raft designed to enable multiple tests of both FCCs and anti-corrosive coatings. The center is located in Hundested Harbour, in the northern part of Zealand in Denmark. Biofouling at the CMTC is seasonal due to the coastal climate in the temperate climate zone, with water temperature around 5–11 °C during spring. The seawater pH ranges from 7.5 to 8.0 and salinity from 14 to 20 ppt [37]. At the CMTC, the performances of FCCs are tested using acrylic panels of standard sizes (200 × 100 mm${}^{2}$) that fulfill the minimum requirements of the 100 cm${}^{2}$ test area established by the European Council of the Paint [38]. The panels are fixed in static frames in three vertical zones: atmospheric zone, splash zone, and fully immersed.

**Figure 1:**
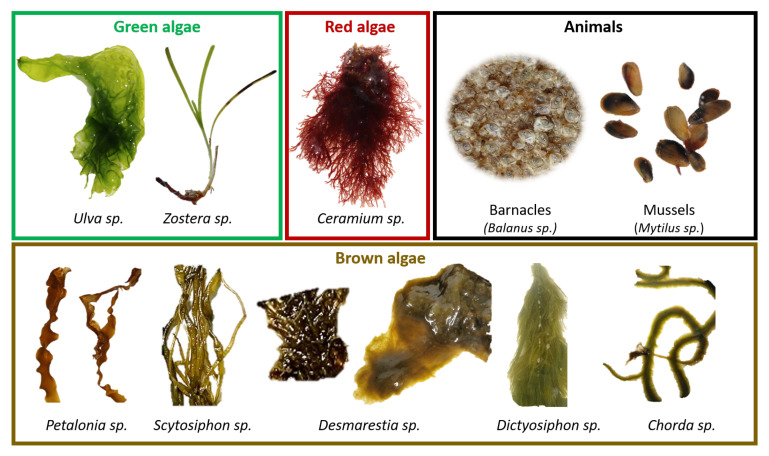
Biofouling species collected in Hundested and used to construct a spectral library. The species were labeled using a reference catalog [39] and inspection under a digital microscope.

To construct the spectral library, we collected samples of the most commonly observed algae and animals for the period (early April) and in the geographical region. In total, nine different species of algae and two types of animals were collected and transported to the laboratory in boxes with seawater. The samples were firstly classified on a species level using a reference catalog [39] and a digital microscope. Afterward, the samples were measured with the HSI system to obtain the optical signatures. All the previous measurements of biofouling were made in vivo and ex situ (i.e., in the lab) on the same day. The collected and classified biofouling specimens are shown in Figure 1.

All the macroalgae can be grouped into three color classes (red or *Rodophyta*, green or *Chlorophyta*, brown or *Phaeophyceae*), each one of these with distinctive photopigmentation and, consequently, distinct reflectance and fluorescence spectrum [40]. Hence, the macroalgae species were grouped according to the colored frames in Figure 1 as green algae (*Ulva* sp., *Zostera* sp.), red algae (*Ceramium* sp.), and brown algae (*Petalonia* sp., *Scytosiphon* sp., 2 different subspecies of *Desmarestia* sp., *Dictyosiphon* sp., and *Chorda* sp.). Two different approaches for the classifications of the algae were considered: a coarse grouping (grouping algae into color classes); a fine grouping (identifying algae at the species level). The collected animals were *Balanus* sp. (barnacles) and *Mytillus* sp. (mussels), and were considered separately in both approaches.

Optical signatures of the standard acrylic panels were also obtained to allow the detection of non-fouled/clean surfaces. Multiple panels were coated with different types of paints and subsequently measured. Some panels had commercial underwater primers with flat gray appearances, whereas others were coated with commercial polyurethane paint (red, blue, white) with a partially glossy finishing.

### 2.2. Sensor

Since the panels are exposed to seawater in static underwater positions at the test site, we decided to develop a staring-type HSI system with wavelength scanning performed by a LCTF. This choice simplifies the setup, acquisition, and post-processing when compared to widely spread push broom systems, thereby allowing the implementation of a more robust system with no mechanical moving parts. Furthermore, a system with no moving parts is more robust to external sea conditions [35].

**Figure 2:**
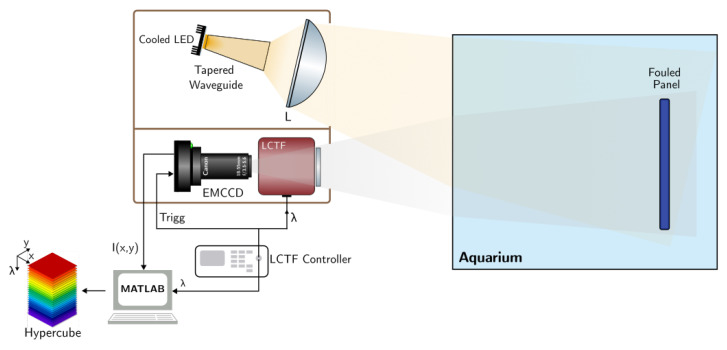
Schematic diagram of the developed hyperspectral imaging (HSI) system. A light-emitting diode (LED) was mounted on a cooling plate and coupled to a hollow tapered waveguide for light mixing and angle-to-area conversion. The uniform output of the waveguide was collected and re-imaged by an aspheric condenser lens (L) onto a coated panel mounted inside an aquarium filled with water. An electron-multiplying charge-coupled device (EMCCD) imaged the panel through a spectral-resolving and electrically-controlled liquid crystal tunable filter (LCTF). The hypercube was reconstructed using a custom computer program by stacking the wavelength-indexed 2D images.

A schematic diagram of the developed system is shown in Figure 2. The system operates in the visible range, from 420 to 730 nm, thus matching the optical transmission window of water [34]. The system is subdivided into two subsystems: an imaging spectrometer and an LED light source. To minimize the influence of background light, the system is enclosed inside a box, with a hard board separation between the two subsystems to minimize the direct coupling of stray light into the imaging segment.

#### 2.2.1. Imaging Spectrometer

The hyperspectral imager is a staring-type system with spectral splitting accomplished by a factory-calibrated LCTF (Thorlabs, KURIOS-XL1/M). The LCTF was built from a stack of alternating polarizers and birefringent liquid crystal plates with electronically-controlled retardation that defines the transmitting wavelength band [41]. The filter has an operating range from 420 to 730 nm with the wavelength-dependent full-width at half-maximum (FWHM) increasing monotonically from around 6.8 to around 14.4 nm. The center wavelength (CWL), λ, is adjustable through voltage in steps of 1 nm by an external controller connected via USB to a computer, with switching times below 5 ms between adjacent wavelengths. By design, the filter transmits polarized light and thus transmission losses are minimized when the polarization of incoming light is parallel to the transmission axis. In our system, the incoming light is mostly unpolarized, inducing an added power loss of about 50%. In this case, the transmission increases with CWL starting from around 0.72% to around 23.6% at the upper wavelength limit. To avoid the use of a relay optical system or a large focal length lens (narrow FOV), the filter was mounted on the object space (i.e., in front of the lens). A commercial camera objective lens (Canon, EF-S 18–55 mm) collected the photons scattered at the surface of an object (panel or biofouling). A low pass filter with a cut-off of 750 nm (Thorlabs, FESH0750) blocked out-of-band infrared light.

To mitigate the effect of chromatic aberrations without focus adjustment, we opted to set the lens with an f-stop of f/10.0. This allowed extending the depth of field without considerable degradation of the transverse resolution but at a cost of a decreased signal. Moreover, since propagation occurs in water, the depth of field was further increased by a factor $n_{w}\approx 1.33$ ($n_{w}$ being the refractive index of water), further reducing the influence of chromatic effects on image quality.

To compensate for the low transmittance of the LCTF, the wavelength-indexed images, *I*(*x*,*y*,*λ*), were formed on a highly-sensitive monochromatic Electron Multiplying Charge-Coupled Device (EMCCD, Oxford Instruments, Andor Luca S). The sensor was cooled down to −20 °C to minimize the dark current, and a real electron multiplication gain of G = 20 was employed throughout all the measurements, as it was found to ensure a good compromise between amplification (faster acquisition) without substantial SNR deterioration from amplified dark current noise. The camera has a 2/3′ format sensor with a 658 × 496-frame resolution and 10 μm × 10 μm pixel size. A 14-bit radiometric resolution alongside a 26,000 e${}^{-}$ pixel well depth were pivotal to accommodate reflectance spanning across high dynamic ranges in a single image without saturation or underexposure [41] and, thus, a more accurate image depiction [26].

#### 2.2.2. Led Illumination System

The evaluation of biofouling levels in underwater surfaces usually occurs in optically shallow waters, both when inspecting ship hulls or fixed panels in a test site. In these circumstances, a passive system is feasible using solar illumination, although the acquisition speed and SNR become highly dependent on the ambient lighting conditions. To avoid this and also compensate for the strong attenuation by the tunable filter, we decided to develop an original high-power light source tailored for our specific application.

**Figure 3:**
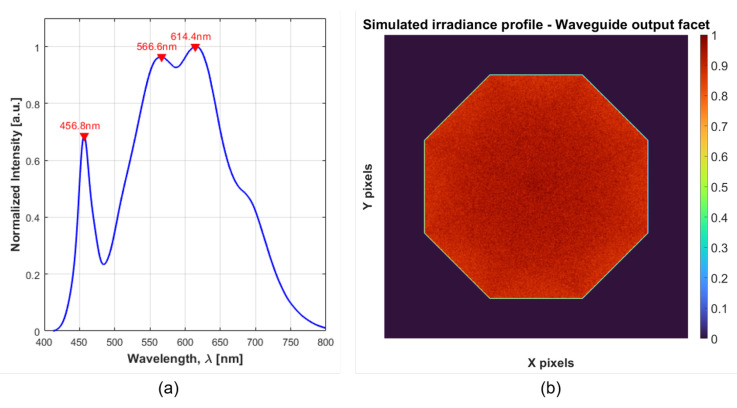
LED emission profiles. (**a**) Measured spectrum of the LED lamp driven at I = 1.5 A, which covers most of the visible wavelengths, thus overlapping with the LCTF transmission range. (**b**) Normalized irradiance at the output facet of the octagonal tapered hollow waveguide as simulated in ZEMAX ($5\times 10^{7}$ analysis rays, 1000 × 1000 pixels detector with 38 mm × 38 mm spatial dimensions, at 0 mm from taper output, smoothing = 2). The Lambertian LED profile was mixed through multiple internal reflections, generating a uniform irradiance pattern at the output facet.

A white LED matrix (Cree, XLamp CM2550 4000 K CRI = 80) was chosen as the emitting element owing to high conversion efficiency, high power, a small footprint with a high fill factor, low cost, and extended stability. The LED was driven at 1.5 A/35 V and the corresponding emission spectrum (Figure 3a) covered the full operating range of the LCTF. The nominal unpolarized integrated flux was around 7700 lm (@ 85 °C), emitted from a circular surface of 19 mm diameter and over a 115° viewing angle (near-Lambertian). The chip was mounted on a cooling aggregate (Fischer, LAM 5 100 24, 0.27 K/W) using a conductive thermal paste to ensure a steady operation point.

To avoid local degradation of the image quality arising from non-uniform irradiance profiles [35] and to maximize power throughput to the panels, the LED was coupled to a 100 mm-long tapered hollow waveguide with an octagonal cross section of side 10 mm at the input faceted and an inclination angle of around 2.5°. The internal walls were coated with highly-reflective polymeric foil (3M, DF2000MA) and the input light was mixed through multiple internal reflections to generate a spatially uniform irradiance profile at the output facet (Figure 3b). Furthermore, the change in cross-section resulted in partial angle-to-area conversion [42]. Since the output light-emitting area increased, the projected solid angle emerging from the light pipe decreased [43]. This geometry allowed to relax the requirements for numerical aperture (NA) of the condenser lens required to image the uniform near-field profile into the object plane located at the far field. An aspheric condenser lens (Thorlabs, ACL7560U-A, D = 75 mm, NA = 0.61) was used for this task. Through adjustment of the lens position along the optical axis, the imaging distance was adjusted to obtain maximum uniformity and irradiance at the plane containing the target.

**Figure 4:**
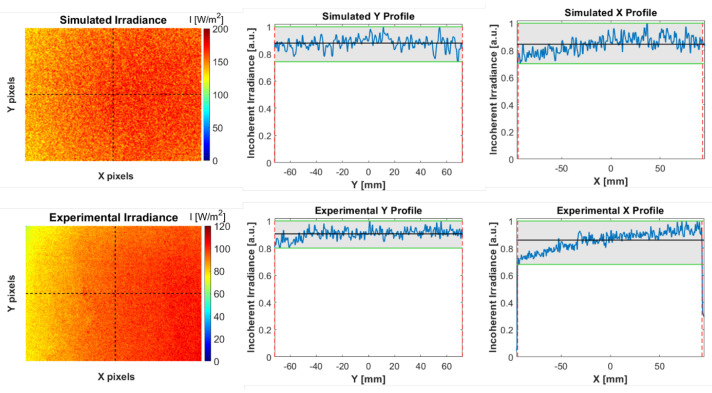
Intensity profiles at the target plane. On top, the ZEMAX simulations under conditions mimicking the experimental setup: d ≈ 1 m from the back vertex of the condenser, 191 mm × 144 mm field of view (FOV), 656 × 498 pixels, $5\times 10^{7}$ analysis rays. On the bottom, the experimental results obtained with a spatially and spectrally uniform target. The decreasing irradiance along the horizontal (X) direction of the FOV was a result of the 15° tilt between LED and imaging system. Noise in the top-hat section of the simulated profiles was a consequence of discrete ray tracing.

The irradiance map at the surface of the panel was evaluated experimentally by imaging a spatially and spectrally (i.e., color-neutral) uniform target through the system without the LCTF. The target was positioned approximately one meter away from the condenser lens back vertex to completely cover the camera frame while fitting the LCTF FOV. The power was evaluated at the center of the frame with a power meter (S120C, Thorlabs), and was extrapolated considering the relative intensities recorded in the frame. The results are shown in Figure 4 alongside the normalized transverse profiles across the center of the frame. The simulated profiles obtained in ZEMAX under the same conditions (distance to panel, detector size, and resolution) are also shown for reference.

To ensure optimal illumination of the panels, the lamp needed to be aligned with the camera FOV. Considering the lateral offset between the camera and LED (bistatic system), combined with the relatively short imaging distances, the light source was tilted at an angle of around 15° relative to the imaging spectrometer. This resulted in an inherent non-uniformity in the illumination across the horizontal (X) direction as the intensity follows an inverse-square law with distance to the radiation source (decreasing intensity towards the left of the frame). Nonetheless, we observed that the vertical profile was extremely uniform. The estimated spectrally and spatially integrated power on the panel was 2.95 W from the experimental map, compared with 4.26 W obtained through simulation. The discrepancy in both total power and peak irradiance likely arises from coupling losses and propagation losses in the waveguide, which were not modeled in the simulation.

### 2.3. Data Acquisition

The acquisitions were performed in a controlled environment with little artificial background lighting so that the LED was the dominant light source. A cubic glass aquarium with a 400 mm side was filled with water and the panels were mounted using a clamping system perpendicularly to the optical axis of the camera and close to the back glass wall of the aquarium to maximize the optical path in the water. The aquarium was slightly tilted to prevent specular reflections at the air-glass-water interfaces from coupling directly into the imaging system. The distance from the camera body to the panels was d ≈ 1.1 m, while the distance from the aspheric lens output vertex was d ≈ 1 m. The panel distance was set by both its size and the maximum acceptance angle of the filter of ±6°, considering the additional magnification induced by the refractive index mismatch at the flat air-water interface (FOV reduction by a factor of $n_{w}\approx 1.33$). The objective lens was adjusted to a focal length f ≈ 40 mm to maximize the panel size on the camera frame. For this focal length, there was no visible vignetting by the filter due to its large clear aperture (CA, 35 mm). Both the LED and the camera were focused accordingly. The spatial resolution was estimated at around 290 μm using a graduated target and the total spatial FOV was 191 mm × 144 mm, roughly matching the size of the panels.

The LCTF CWL was scanned in the full 420–730 nm range in 5 nm sampling steps, originating hypercubes with 63 channels in total. As each wavelength band was acquired in a single shot, the exposure times were individually adjusted for each channel to compensate wavelength-dependent responses (e.g., LCTF transmission, water absorption, LED spectrum), and thus improve the overall SNR while preventing under and overexposure. This was crucial to safeguard good spectral reliability [44]. The images were captured using the Andor SOLIS software and the total acquisition time was approximately 6.6 s under the conditions reported herein. The acquisition time was mainly limited by the exposure times at the extreme bands, namely λ<440 nm and λ>700 nm. For λ<440 nm, the exposure time was set by a combination of low LCTF transmission and low LED irradiance; for λ>700 nm, by a combination of stronger absorption in water, low LED irradiance, and lower camera responsivity. For this reason, and since the SNR scales approximately with the square root of the exposure time [41], the exposures at the edges were comparatively much larger than at the center bands. For reference, the exposure time employed at λ=430 nm was 500 ms, while 50 ms at λ=500 nm, 15 ms at λ = 600 nm, and 250 ms at λ=730 nm. The resulting spectrum was, in practice, the convolution between the instrument response function and the specimen’s actual reflectance spectrum. Due to the finite size of the LCTF transmission curves at each CWL setting, one expects blurring of the spectral features that can be partially corrected in post-processing through deconvolution. For each channel, the same exposure was used throughout all measurements.

### 2.4. Spectral Data Processing and Analysis

**Figure 5:**
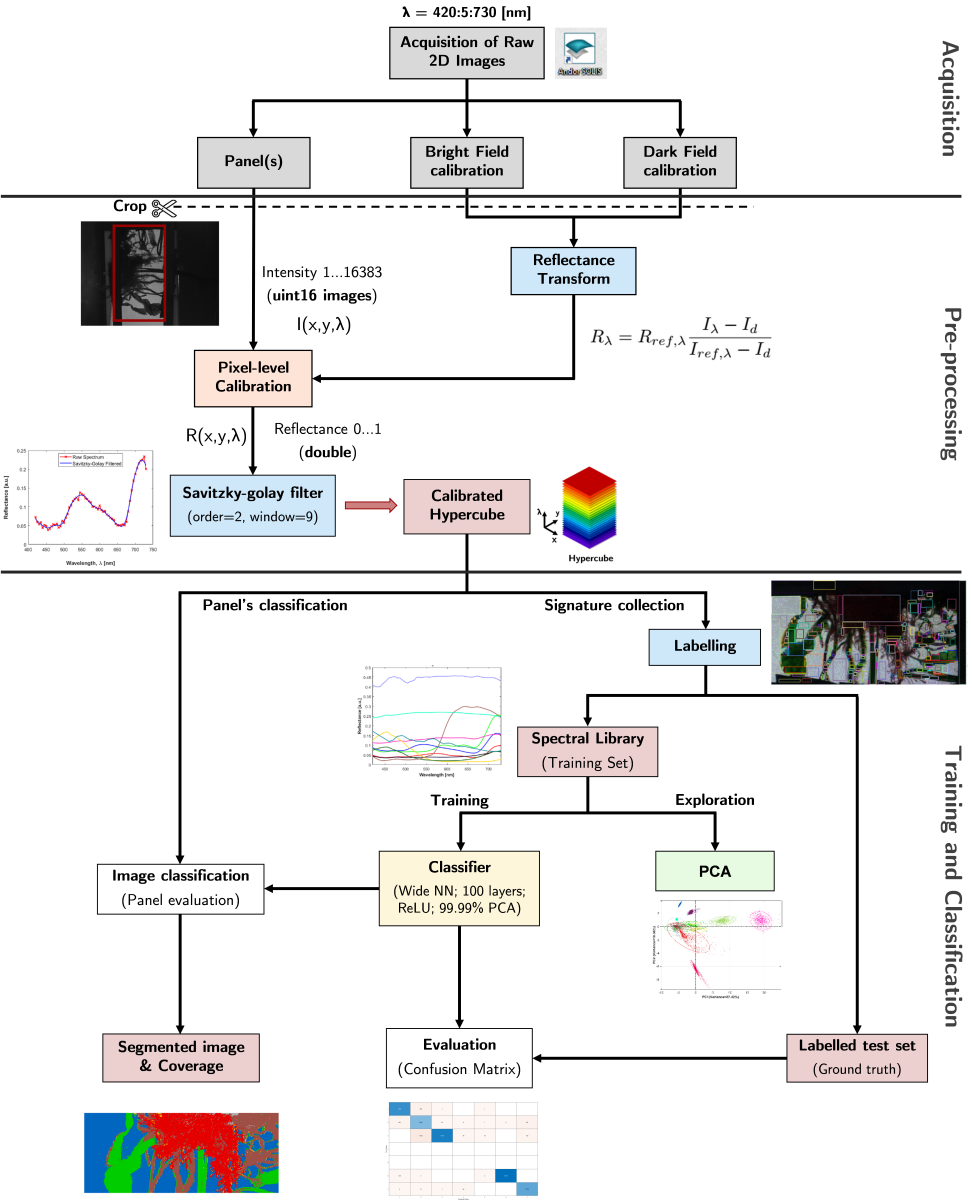
Workflow for pre-processing, training of the model, and classification of fouled panels.

After acquiring a full stack of 2D images indexed to the respective wavelength, the data were processed in MATLAB with custom-built scripts. A simplified diagram representing the workflow is presented in Figure 5. In the first stage, pre-processing was applied to crop the regions of interest (ROIs), convert intensity data to reflectance, and smooth the obtained spectra. Afterward, a classifier was built using a supervised approach with a spectral library as the training set. The classification accuracy was then evaluated using a previously annotated test set as ground truth. The classifier could finally be applied to a new calibrated hypercube to estimate the coverage of each biofouling class on the surface of a panel.

#### 2.4.1. Pre-Processing and Reflectance Transform

The raw value obtained by a camera is a digital number that captures the recorded photons and dark current over the integration time [45]. The intensity, *I*, at a pixel location (*x*,*y*) in the monochromatic sensor and at a particular CWL setting of the LCTF, λ, can be described as a function of the system and medium parameters (adapted from [41,46]):

(1) $$I\left(x,y,\lambda \right)=t_{exp}\left(\lambda \right)\left(I_{d}\left(x,y\right)+\int_{0}^{\infty }I_{s}\left(x,y,\lambda^{\prime }\right)e^{-2\alpha \left(\lambda^{\prime }\right)d_{w}}R\left(x,y,\lambda^{\prime }\right)\Gamma \left(x,y\right)\tau_{TF}\left(x,y,\lambda^{\prime },\lambda \right)\tau_{O}\left(\lambda^{\prime }\right)ℜ\left(\lambda^{\prime }\right)\;d\lambda^{\prime }\right)$$

where $t_{exp}$ is the exposure time, $I_{d}$ is the dark current, $I_{s}$ is the light source spectral radiance, α is the wavelength-dependent attenuation coefficient of water, $2d_{w}$ is the total optical path length in water (two-way propagation), *R* is the spectral reflectance at the surface of the target, $\tau_{TF}$ is the transmission function of the LCTF at the CWL λ, $\tau_{O}$ is the transmission of the objective lens, and *ℜ* is the spectral responsivity of the camera. A function Γ(x,y) is introduced to represent the optical transformation between coordinates in the panel plane and coordinates in the imaging space, which includes refraction at the air-glass-water interfaces. Although the integral is performed over all wavelengths (variable $\lambda^{\prime }$), the spectral confinement is implicit both in the LCTF transmission function and the sensitivity range of the camera. This description is valid under the following assumptions: all parameters are constant over a time interval corresponding to the camera exposure time; there is no extinction in the optical path in the air; the underwater optical path is approximately the same in both directions, $2d_{w}$, and absorption is homogeneous (i.e., no spatial dependence); the lenses transmission is constant over its CA as well as the responsivity across the photosensitive area of the camera; contribution from diffuse and Fresnel reflections from the air-glass-water interfaces is negligible (tilted interface).

To reliably use a hypercube for hyperspectral analysis and to make it externally consistent [36], an accurate estimation of the surface spectral reflectance is required. This quantity is an intrinsic property of the objects and it is independent of the illumination source, properties of intervening water volume, and imager parameters. Therefore, this quantity can be used for global comparison. The reflectance values, R(x,y,λ), were recovered from the raw intensity signal, I(x,y,λ), using a two-point calibration procedure to cancel out the system and medium parameters in Equation (1). Ideal lighting conditions are commonly assumed [47], for which the measured irradiance from an object is considered independent of its position within the FOV. However, in the presented system, the tilt in the LED relative to the camera/target induces spatial inhomogeneities at the imaging plane. Furthermore, the filter transmission is not uniform across its CA. As a consequence, a flat field correction was required. This pixel-wise correction ensures that differences in reflectance were solely a result of local differences in the surface properties of the target and that all pixels were directly comparable.

Calibration was performed using a reflectance standard with well-known properties. In this case, a calibrated Lambertian reflectance target with a spatially and spectrally homogeneous diffuse reflectance of $R_{ref}\approx 10\%$ was used (SphereOptics, RTI-010, 200 mm × 250 mm) that fills the full FOV. Three full hypercubes must be acquired at the same exposure time [45]: a hypercube of the scene to transform into reflectance values, I(x,y,λ); a hypercube of the reference standard (bright field cube), $I_{ref}\left(x,y,\lambda \right)$; a dark current hypercube (dark field cube), $I_{d}\left(x,y,\lambda \right)$, obtained by blocking the incoming light completely. Both the reference standard and the fouled panels were submerged at the same imaging plane. The transformation from camera counts to reflectance is then determined by:

(2) $$R\left(x,y,\lambda \right)=R_{ref}\left(\lambda \right)\frac{I\left(x,y,\lambda \right)-I_{d}\left(x,y,\lambda \right)}{I_{ref}\left(x,y,\lambda \right)-I_{d}\left(x,y,\lambda \right)}$$

assuming that all surfaces behave as Lambertian scatterers [26]. The position-specific transform models described by Equation (2) were computed at pixel level from the same location in each of the three cubes [36].

**Figure 6:**
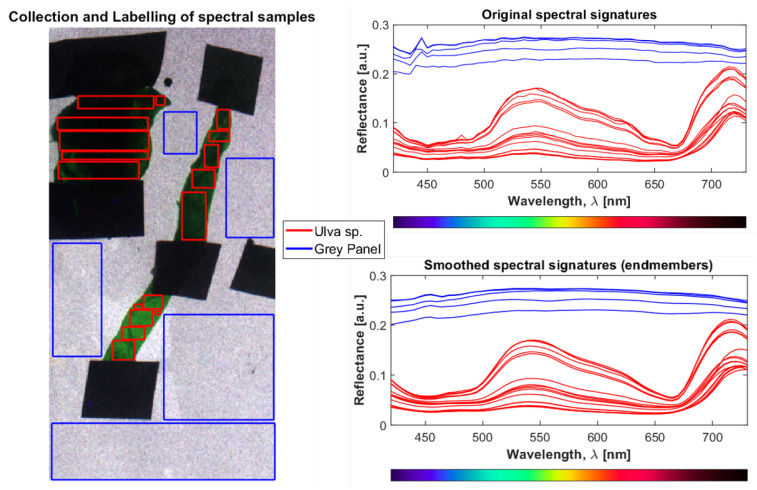
Example of labeling procedure for *Ulva* sp. and “gray panel” samples. On the left, annotated pixels selected from rectangular regions of interest (ROIs) of the hypercube. On the right, the raw reflectance spectra and respective smoothed spectra. For representation simplicity, each curve represents the average within a ROI, although all enclosed the individual pixel signatures were added to the dataset. The reduction of noisy features through low pass filtering is noticeable (e.g., gray panel at 450 nm); broadband spectral features are preserved. Intra-species variability is visually noticed when comparing the dense multi-layer patch on the top left with the light green section in the center.

After calibration, the reflectance spectra of the pixels were low-pass filtered using a second order [48] Savitzky–Golay filter with a window size of nine channels (i.e., 45 nm) [49] to smooth the signals without substantially changing the fine spectral features (Figure 6). All subsequent processing was performed on the smoothed, cropped, and reflectance-transformed hypercubes.

#### 2.4.2. Signature Collection (Training Set)

For supervised classification tasks, a set of known and properly labeled optical signatures is required as an input to train the algorithm and produce a classifier that can be applied to new data a posteriori. For this purpose, a spectral library (end members) was built by independently acquiring the hypercubes for the typical biofouling specimens collected from the CMTC. The samples were first identified, to assign a correct class and build the predictor, and then attached to a panel to be measured by the HSI system. Since each pixel contains a full reflectance spectrum, the information in each hypercube amounts to tens of thousands of spectra. Therefore, from the calibrated hypercubes, several ROIs containing multiple pixels were selected for each specimen to account for intra-species variability (e.g., due to differences in density, orientation, development, and health status), obtaining a more robust dataset. Similarly, several pixels were gathered for the coated panel classes to account for surface heterogeneity. All enclosed pixels were then annotated with a class label and added to the library. The annotation process underlying the construction of the spectral library is shown in Figure 6. The total number of spectra in the library were collected from a total of 16 hypercubes and amounted to 438,657, divided between the 9 different algae species, the two animals, and the 6 differently-colored panels. Detailed information about the sample distributions among classes is provided in Table S1 in Supplementary Material.

#### 2.4.3. Principal Component Analysis

Prior to the training of the neural network, an exploratory PCA [50] step was performed on the collected reflectance data to assess spectral similarities and differences between the selected classes. PCA is a multivariate statistical analysis that performs a linear transformation of the spectral signatures, so that most of the variance in the dataset is captured in the first principal components (PCs). This step allows for reducing the dimensionality and complexity of the dataset. Assuming that neighboring bands are usually highly correlated and yield redundant information about the scene [51], redundancies could be discarded by selecting the most important spectral features among the 63 variables/wavelength channels. This way, the total amount of data available for training of the classification model and, consequently, the processing times could be reduced. For representation purposes, a total of N = 2000 data points were randomly selected within each class (with the exception of the barnacles for which the total number of samples was considered). PCA was then applied to the standardized data (mean-centered and with normalized variance) and the results represented in biplots.

#### 2.4.4. Classification: Neural Network

A classifier was trained using supervised machine learning to map an input spectrum to a specific biofouling class [21]. This group of algorithms learns and trains from a correctly labeled dataset to predict outcomes on unforeseen datasets, i.e., in newly measured panels. The constructed spectral library was used as a training set paired with the corresponding labels as desired response values.

In this work, we employed a wide neural network (WNN) for the classification task. When large training sets are available, as in this case, neural networks can yield powerful representation capabilities and prevent overfitting [52], while showing superior accuracy compared to SVMs [53]. The training was performed using the "Statistics and Machine Learning Toolbox" in MATLAB [54], more specifically the "Classification Learner" app. The model parameters were set as follows: single hidden layer with 100 neurons; ReLU activation function; regularization parameter λ=0; standardization of data active; five-fold cross-validation. We limited the dimensionality of the data using PCA to reduce the training time [53] and overfitting, thus improving the model generalization to new data. Only the PCs explaining 99.99% of the variance were considered, which corresponded to 18 out of 63 features. In these conditions, the accuracy from cross-validation within the training dataset was around 99%. Hyperparameter optimization was left outside the scope of this publication.

After training the model, the resulting classifier could then be applied to a new hypercube to assign a unique class to each pixel and produce a segmented image. Subsequently, the coverage percentage of each biofouling class could be evaluated. Because PCA is applied prior to training, the new hypercube samples need to be transformed into the same PCA space prior to classification.

#### 2.4.5. Testing and Classification Accuracy

**Figure 7:**
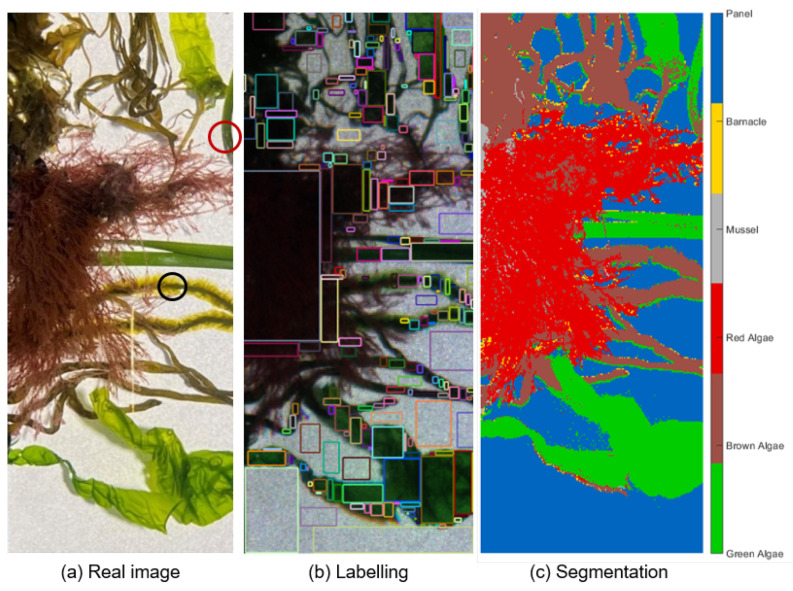
Image of the model target constructed by attaching biofouling collected at the CoaST Maritime Test Centre (CMTC) to a panel coated with a commercial underwater primer. In (**a**), the RGB image where two algae from different groups are encircled (brown in black circle, green in red circle) to demonstrate that different classes can sometimes depict similarities in color. In (**b**), the labeling of the measured hypercube to generate the ground truth. In (**c**), the segmented image resulting from the application of the wide neural network (WNN) classifier to the hypercube.

The model’s capability of predicting new and unseen input data was tested using a dataset comprised of spectral samples that have not been used for training and collected from a spatially disjoint target (i.e., hypercube) to avoid the possible impact of correlated neighboring pixels on the results [55]. The performance of the WNN classification model was evaluated using accuracy as a figure of merit. The test dataset was externally collected from a model target constructed by attaching several biofouling specimens collected at CMTC to a coated panel (Figure 7). A total of seven different types of algae, similar to the species used for training (but different samples) were utilized: two green, one red, and four brown algae. The algae species were *Ulva* sp., *Zostera* sp., *Ceramium* sp., *Petalonia* sp., *Scytosiphon* sp., *Desmarestia* sp., and *Chorda* sp. (see Figure 1). Due to shape factors, no mussels were included in the model target as its attachment proved impractical. Likewise, no barnacle samples were added due to their strong adherence to a surface, which made it impractical to transfer them to the model target. A test set of 41,502 spectra was sampled. Its distribution is disclosed in Table S1 in Supplementary Material.

The obtained calibrated hypercube of the artificially generated model target was labeled using the same procedure as in Section 2.4.2 (Figure 7b) to generate the ground truth against which the classes predicted by the classifier could be compared to generate a confusion matrix and evaluate the overall accuracy. The latter was calculated by dividing the trace of the matrix by the sum of its elements (total number of samples). For each class, the true positive rate (TPR), or sensitivity/recall, was calculated as the ratio between the number of correct classifications (diagonal element, or true positives) and the total sample points of the class in the ground truth (sum of the corresponding row, i.e., of true positives and false negatives). The false negative rate (FNR) was determined by FNR(%)=100−TPR(%). The two previous values are presented to the right of the confusion matrix. The positive predictive value (PPV) or precision for each class is presented below the error matrix alongside the false discovery rate (FDR). The former is the ratio between the true positives and the sum of the true positives and false positives for the considered class, i.e., the sum of the respective column of the confusion matrix. Similarly, the FDR is related to the PPV through FDR(%)=100−PPV(%).

#### 2.4.6. Evaluation of Real Fouled Panels

To test the developed method on a real case scenario with intergrown biofouling, the classifier was evaluated on two coated panels that had been exposed at CMTC for a period of more than a year. Both panels were coated with a white commercial polyurethane paint with no fouling control properties and were fully covered with algae, mussels, and barnacles. As the panels were heavily fouled, we decided to evaluate them for different simulated states of biofouling. We started by acquiring the hypercube for the fully fouled panels, and then gradually scrapped some of the fouling to reproduce different degrees of fouling and to expose some of the underlying coated panel. Three states were measured: fully fouled, medium-fouled, and low-fouled panels. As the biofouling on the panels was intergrown, the WNN classification was only evaluated qualitatively through direct comparison with a visual inspection of the corresponding color images.

## 3. Results

### 3.1. Spectral Library

**Figure 8:**
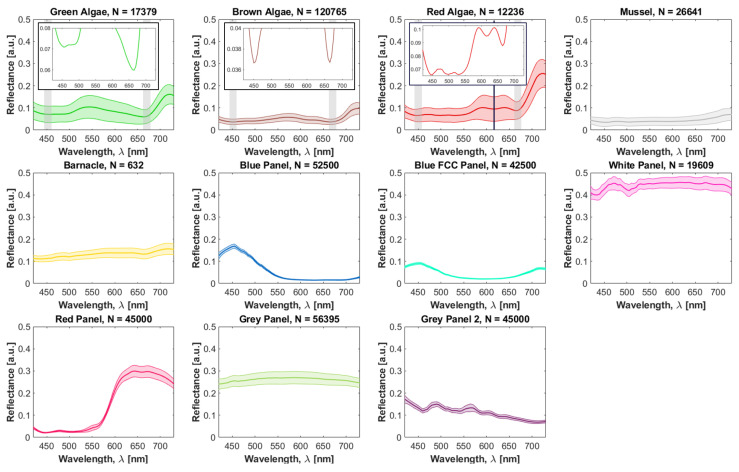
Mean signatures from the spectral library used as the training set for the WNN model with the coarse groups of algae. The shaded regions represent the standard deviation. For the algae signatures, the vertical gray bars indicate chlorophyll-a (chl-a) absorption bands; for the red algae, the blue vertical line indicates the in vivo satellite band absorbance peak of chl-a. For the latter classes, an inset is also shown with a zoom-in on local absorption minima. The total number of pixels sampled per class is disclosed in the title of each subplot.

The mean spectra for the coarse groups of the fouling classes are shown in Figure 8. The total number of pixels spectra averaged per class is disclosed in the title of each subplot. The shaded areas represent the standard deviation.

All algae specimens are photoautotrophs and some optical fingerprints in their spectra are inversely related to absorption by light-harvesting pigments [56]. Two local reflectance minima were prominent in all the green, brown, and red spectra at 450 ± 10 nm and 665 ± 15 nm (highlighted by the vertical gray bars). These features coincide with the in vivo peak absorption of chl-a, a primary photosynthetic pigment present in all these organisms, and are consistent with past measurements on the red [26] and green [49] algae. Moreover, an additional weaker minimum was observed at 615 ± 15 nm in red algae previously linked to a satellite band absorbance peak of chl-a [57]. The reflectance peak observed at 720 ± 15 nm was a superposition of autofluorescence from chl-a (dominant) [58] and elastic scattering. This peak was more accentuated for red algae likely due to a greater chl-a content. Regarding absolute values, all the algae have a visibly dark appearance and thus relatively low reflectance values across the full spectrum (<16% when the fluorescence peak is disregarded). Green algae exhibit a reflectance peak at 545 ± 10 nm, consistent with their color. Brown algae exhibit a peak at 570 ± 10 nm and a flatter spectrum with lower all-around reflectance. Red algae exhibit two reflectance peaks at 590 ± 10 nm and 640 ± 15 nm. The spectral features and reflectance levels obtained herein are similar to those observed for other red [26] and green [40,59] algae, indicating an accurate calibration procedure.

Color-neutral organisms, particularly mussels, barnacles, and white/gray panels, have a relatively constant reflectance across all wavelength channels. As expected, the white panel samples show higher reflectance values among all the endmembers (around 45%), while mussels constitute the lower-reflecting object (≈4%). A slight increase in reflectance from mussels was observed as wavelength increased, linked to the slightly brownish color of these organisms (see Figure 1). Very few barnacle samples were collected (N = 632) due to the lack of isolated samples at the CMTC. For these, we observed a small dip at 665 ± 15 nm and a peak at 720 ± 15 nm that clearly signal the presence of chl-a in the collected spectral samples. This might be an indication that the barnacles were either mixed with algae (e.g., on their surface) or the presence of large chl-a content in the aquatic medium that distorted the real signature. The remaining panels had broadband reflectance peaks at the wavelengths that match their colors: 455 ± 10 nm for both blue panels, and 645 ± 15 nm for the red panel. The blue FCC panel also had a local maximum at 720 ± 15 nm. Since this was a commercial FCC, its formulation is undisclosed and the origin for this peak was unclear. We speculate that it stems from additives in the coating.

### 3.2. Principal Component Analysis

**Figure 9:**
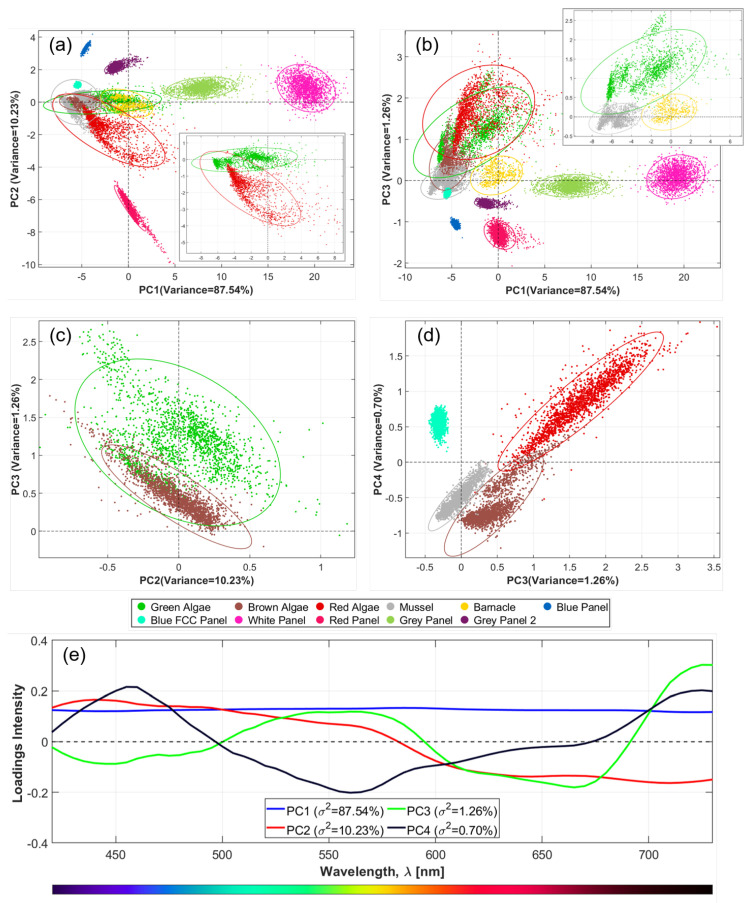
Principal component analysis biplots (**a**–**d**) and loadings (**e**) of the first four principal components (PCs). The displayed biplots are, in order: (**a**) PC1 vs. PC2, (**b**) PC1 vs. PC3, (**c**) PC2 vs. PC3, and (**d**) PC3 vs. PC4. The ellipses represent the class-specific 95% confidence interval, with the longest axis along the direction with the largest variance within each group [47].

The PCA results are displayed in Figure 9. Biplots with the 95% confidence ellipses for each class are presented alongside the loadings for the first four PCs that explain, 87.54%, 10.23%, 1.26%, and 0.70% of the total variance in the spectral signatures within the dataset. PCA highlights spectral differences and similarities among samples. In the biplots, point clusters indicate close spectral relations, and non-overlapping ellipses might be interpreted as spectrally-distinguishable groups [47]. The loadings quantify the importance of each independent variable, i.e., each wavelength channel, to each PC: larger loading values indicate a larger relative importance of a specific wavelength to the considered PC, with negative values meaning a negative correlation. To support the discussion, we only show a selection of the biplots that better demonstrate spectral differences among classes. On the bottom two biplots, only the classes that are not distinguishable on the top two are shown, to facilitate intelligibility.

In general, the PCA biplots disclosed that most classes had distinct optical signatures and could be mostly discriminated based on reflectance spectra alone. All the panels were completely distinguishable from one another and from the fouling classes when PC1 was plotted against PC2 (Figure 9a), with exception of the “Blue FCC Panel” that overlaps with the confidence ellipse of “Mussels”. Nonetheless, the two were completely distinguishable when PC3 was plotted vs. PC4 (Figure 9d). This was a valuable indicator since one of the key tasks for a biofouling monitoring system is to discriminate clear (panel exposed) from the biofouled surface.

Barnacles and mussels were also spectrally distinguishable from the other classes, with the exception of green algae. However, by plotting PC1 vs. PC3 for only these three classes (inset in Figure 9b), one observed that, although the ellipses overlapped, the datapoints did not. This meant that the three classes would likely be separable to some extent. The underlying reason is that the confidence ellipses are representative of normal distributions. Nevertheless, for the green algae, two different species were sampled. Similarly, for the mussels, both dark and brown specimens were sampled, thus skewing the distributions.

Regarding the algae groups, the spectral similarities were larger, particularly due to the characteristic fingerprints from chl-a in all specimens. Following the same reasoning as before, the datapoints for green and red algae groups have almost no overlap in the PC1 vs. PC2 biplot (see inset in Figure 9a), although there was substantial overlap between the respective confidence ellipses. The ellipses and some datapoints for red and brown algae overlap in all graphs, but the superposition was only slight when PC3 was plotted vs. PC4 (Figure 9d). Among all classes, brown and green algae were expected to introduce the biggest confusion in classification, as there was an overlap between datapoints and ellipses in all the biplots. When considering the first four PCs, the overlap was smallest in the PC2 vs. PC3 biplot (Figure 9c). We observed, in some instances that the brown algae considered in this study had a greenish appearance. For example, the *Chorda* sp. sample region marked by a black circle in Figure 7a was considered brown algae but had a green-like appearance towards the filaments on the edge, resulting in misclassification as green algae in the segmented image. Likewise, the *Zostera* sp. marked by a red circle was a green algae, but some regions were darker than others and were confused with brown algae. This is a possible justification for the spectral similarities between these classes.

Another observation from the biplots was that some of the classes exhibit larger intra-class variation in spectra, reflected in more dispersed cluster of points. In particular, red and green algae were the categories that stood out, also indicated by the broader standard deviations in Figure 8. For the green algae, two species were sampled, but for the red algae only one species constitutes the dataset, so the justification does not lay solely on inter-species differences. This was also a result of the effort to sample ROIs depicting distinct perceived reflectance within each species, to make the classifier more robust to intra-species variability.

Concerning the loadings, for PC1 they were equally distributed across all wavelengths, meaning that all channels carried similar weight. Considering that PC1 has been associated with brightness [26,60], one can hypothesize that reflectance intensities were the main discriminatory feature. As for the remaining PCs, some wavelengths appeared to explain spectral differences among classes more than others. For instance, in PC3 (green curve in Figure 9e) the wavelengths around 550 nm and above 700 nm (chl-a fluorescence) seemed to have a strong positive influence, while the wavelength contribution to the variance seemed to be negatively correlated to chl-a absorbance (peak absorption bands match the location of the minima in the loadings). Therefore, there was a clear indication that the fingerprints associated with chl-a were key to distinguishing between algae and non-algae classes. Further pigment analysis is needed to assess the nature of the PCs since PCA serves only as an exploratory tool [49].

From this analysis, misclassifications were expected between classes that overlapped in the biplots, especially between brown algae and red/green algae. Outliers also curtail classification accuracy. Although the presented analysis was restricted to four PCs, we decided to reduce dimensionality while preserving a number of PCs justifying >99.99% of total variance (18 out of 63 PCs) prior to training because smaller spectral features that could be crucial for classification would be preserved by considering higher PCs. This was particularly relevant for classes demonstrating similarities in the optical signatures.

### 3.3. Classification and Coverage Estimation

#### 3.3.1. Model Target

**Figure 10:**
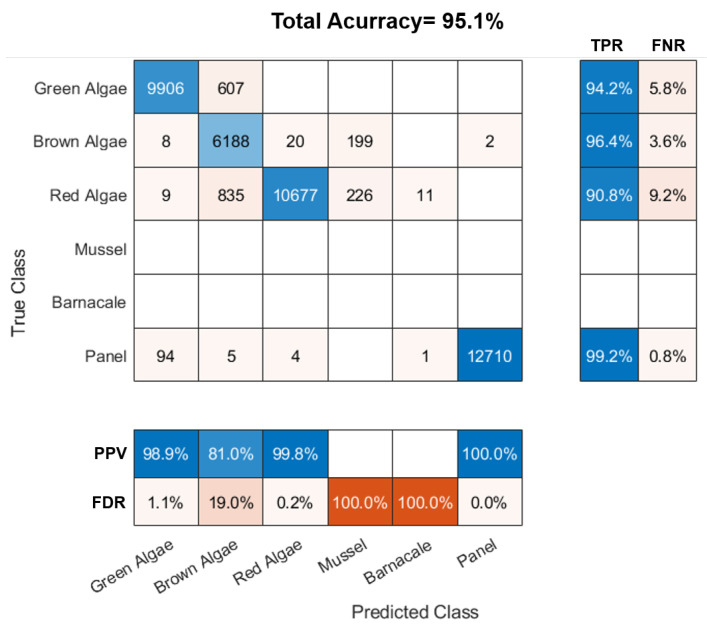
Error matrix for the WNN classification of the model target with ground truth in rows and predictions in columns. The true positive rate (TPR), or recall, and false negative rate (FNR) for each class are presented on the side; the positive predictive value (PPV), or precision, and false discovery rate (FDR) are presented below. No mussels or barnacles were present in the model target.

The classifier performance was assessed by presenting it with unknown (i.e., previously unseen) hyperspectral data acquired using a custom-built model target. The accuracy of the WNN model was quantified by comparing the labeled ground truth (Figure 7b) with the prediction of the model on the test dataset. The segmented image with the unique classes assigned by the trained WNN model to each pixel is shown in Figure 7c. The confusion/error matrix obtained by comparing the true and predicted classes is presented in Figure 10.

The overall accuracy of the WNN classifier in the model target was around 95.1% (see Figure 10). All the differently colored panels were merged under a single class “Panel” for the purpose of classification since, in practice, the central task was not to discriminate panel colors but rather the percentage of clear surface. On top of it, the color of the panels was well-known prior to submersion. The total training time for the model was around 800 s, while the time it took to classify the 430 × 177 pixels segment of the hypercube was around 135 ms (averaged from 500 measurements).

In terms of individual classes, the PPV for the panel category was almost 100% while the TPR was 99.2%. These results disclosed that the panels could be almost completely isolated from fouling with negligible false positive and false negative counts, which is in line with the observations from the PCA. Among the algae classes, the brown algae was the one depicting the smallest PPV at 81%, with 607 and 835 pixels being mistakenly classified as green or red algae, respectively. Its TPR, however, was around 96.4%, with 199 of the pixels being misclassified as mussels. The confusion between brown algae and the other two algae classes had already been signaled in the PCA analysis. For both the green and red algae, the PPV was above 98%, meaning a negligible narrow rate of false positives. The TPR for the green algae class reached an estimate of 94.2%, skewed by the misclassifications as brown algae. Similarly, the TPR for the red algae was 90.8%, suffering from additional false negatives arising from misclassification as mussel. Confusion between red and green algae was negligible, supporting the previous remark in PCA that, although ellipses overlap, the points were mostly distinguishable (non-normal distributions). As mentioned, no barnacles nor mussels were included in the test set as attachment to the model panel was not viable.

**Figure 11:**
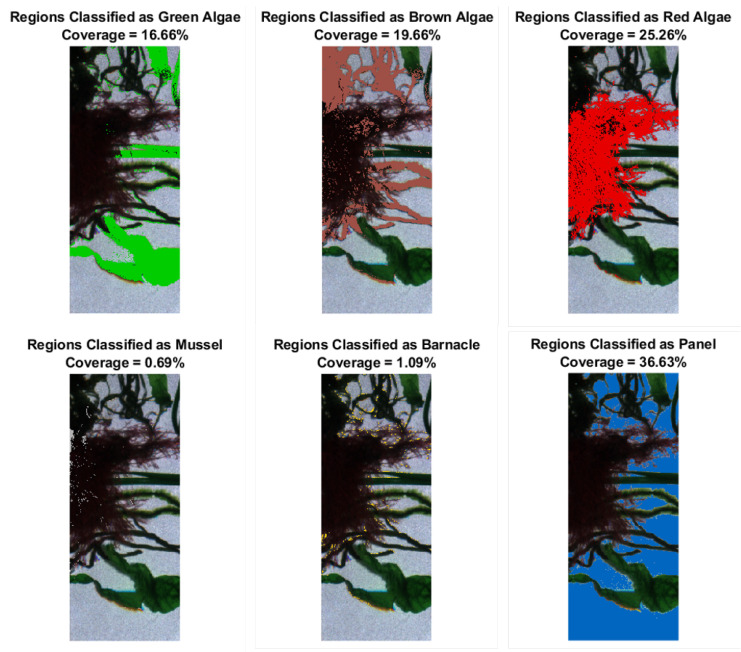
Maps of each category obtained by the WNN classification on the model target, including coverage percentages. The underlying image was obtained by selecting three RGB bands from the hypercube. The overlay indicates the pixels classified as the corresponding class.

For the evaluation of biofouling on a coated surface, knowledge about the coverage percentage of algae and animals, as well as percentage of clean panel are essential metrics. From the classification results obtained with the WNN model, the coverage percentage for the different classes was calculated as the ratio between the pixels in each class and the total number of pixels. The coverage maps for each class estimated by the classifier on the model target are shown in Figure 11. The coverage of the model target was determined to be 16.66% of green algae, 19.66% of brown algae, 25.26% of red algae, and 36.63% of the panel. The WNN model wrongly classified 0.69% as mussels and 1.09% as barnacles. From the segmented image in Figure 7c, one notices that most of the misclassification occurs at the edges of each object. We speculate that this was mainly due to mixed signatures between two overlapping objects, as for instance panel and algae, which changes the compound spectra and creates confusion in the classification step.

To evaluate the impact of dimensionality reduction on the classifier performance, we trained a new model under the same conditions but without applying PCA (i.e., using the 63 available variables). While the total training time increased to around 4800 s, the average classification time slightly decreased to 123 ms since the data no longer needed to be transformed into the PCA space prior to classification. Accuracy-wise, the overall accuracy decreased to around 85.8% indicating overfitting. Therefore, by employing a more restricted dataset size-wise, it was possible to reduce training times and enhance the overall model accuracy.

#### 3.3.2. Fine Classification

A supplementary classifier was trained to ascertain to which extent the model could discriminate algae species even among the same color class and provide a more detailed description taxonomy-wise. A new neural network with the same parameters was trained with training and test sets annotated according to the fine grouping (species labels presented in Figure 1). The samples were collected from the same ROIs. The new spectral library, confusion matrix, and coverage per species after classification with the new model are presented in Supplementary Material Figures S1–S3, respectively.

The overall accuracy with the new species-specific classifier dropped to 81.5% on the model target. The TPR and PPV for the panel class remained mostly unchanged, as well as those for *Ceramium* sp. (only red algae in the sampled set). On the other hand, there was substantial confusion between algae species within the same color group. For instance, among the green algae, the *Ulva* sp. demonstrated the largest PPV and TPR, at around 80.9% and 91.5%, respectively. Nevertheless, the number of false negatives for the *Zostera* was considerably high and the TPR amounted only to 12.4%. As for the brown algae, similar confusion was observed among the different species. The TPR and PPV peaked for the *Ceramium* sp. at 93.7% and 99.5%. The PPV for all other species was below 50%, meaning that the number of false positives surpassed the number of true positives. The *Petalonia* sp. was overall the species displaying worse classification potential, with a TPR and PPV of only 2.5% and 19.4%, respectively.

These results demonstrate that the species within each group of algae in the current dataset were relatively indistinguishable. This outcome was expected since the classification was based purely on reflectance spectra alone and the species shared common pigmentation and characteristics resulting in very similar signatures. Further information, for instance, morphology, is needed for accurate taxonomic classification at the species-level.

#### 3.3.3. Real Targets

**Figure 12:**
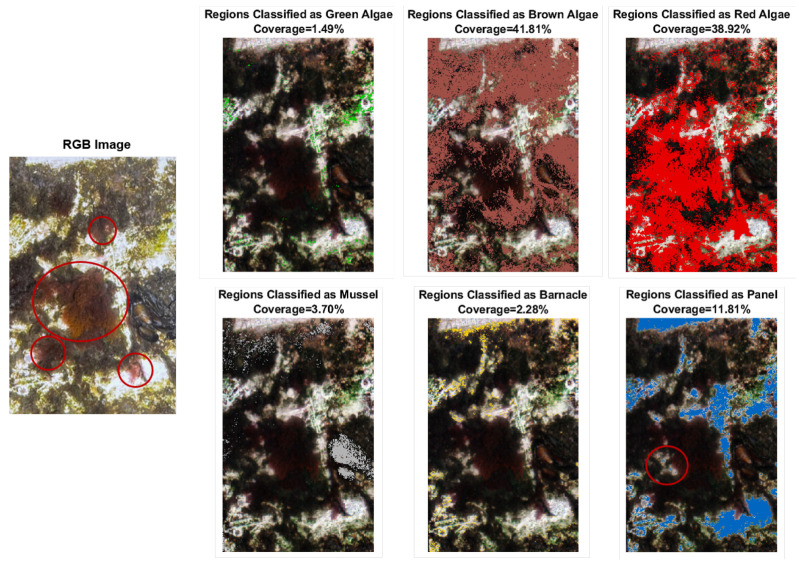
Classification and coverage percentage of biofouling for a medium-fouled panel obtained from using the WNN. On the leftmost side, the RGB image of the panel taken with a commercial camera is shown. Some of the red algae detected under visual inspection of the panel are encircled to qualitatively compare with the classification with the WNN model.

Figure 12 shows the RGB image and respective classification with coverage estimation after application of the WNN classifier to a medium-fouled panel. The trained model estimated that the dominant biofouling groups were brown and red algae, with coverage of 41.81% and 38.92%, respectively. Only around 11.81% of the underlying white panel was exposed. The less prevalent species on the panel were, mussels, barnacles, and green algae covering only 3.70%, 2.28%, and 1.49% of the considered panel surface, respectively. Another panel subject to the same exposure at the CMTC was evaluated and the segmentation results are documented in Figure S4 in Supplementary Material for three distinct states of biofouling.

Direct visual comparison between the RGB and segmented images indicated a relatively reliable and accurate identification of panel pixels. As for the other classes, red and brown algae seemingly dominated the panel surface. Red algae dominated mostly the bottom left portion of the panel, and the more evident specimens marked with red circles in the RGB image were correctly identified. Brown algae were dominant on the top and right, which matches visual observations. Because the algae grow intertwined, some regions were harder to identify with the naked eye. Green algae were mostly present in the boundary regions, and those too were quantified by the model. The two mussels at the center-right of the panel were properly classified. Nevertheless, some pixels at the top were misclassified and should instead be attributed to the brown or red algae class, similar to the observations with the model target. Lastly, the detection of barnacles seemed to be an error in the classification, most likely due to the distorted endmember signature. Although visible traces of prior presence of barnacles were identified, they were not directly present in the imaged panel.

## 4. Discussion and Conclusions

### 4.1. Staring-Type Hyperspectral Sensor

In the present paper, we explored the feasibility of using HSI for biofouling assessment in submerged panels. A staring-type system was implemented using a LCTF as the spectral resolving element motivated by the static nature of the application. The biggest downside of our HSI system was the low LCTF transmittance that led to overall longer acquisition times. As a counteraction, we used an EMCCD alongside a high-power lamp designed to tailor our application. The implementation of an active system also allowed to compensate for extinction due to propagation in water.

A white LED element was chosen as the centerpiece of the illumination system since it met the requirements for high stability, high energy efficiency, a long lifetime, and a small footprint [35]. A novel design combining a tapered hollow waveguide for mixing the emission profile and reduction of the angular spread of the light cone, with a high-NA condenser lens, was used to accomplish high-power and uniform illumination of the panels. A slight non-uniformity arising from the horizontal tilt of the LED lamp was corrected with a flat-field pixelwise calibration step. In the future, a second LED lamp similar to the latter can be implemented symmetrically to correct the non-uniformity and double the irradiance levels (and, thus, reduce the acquisition times). Proper uniform illumination is crucial to avoid local degradation of image quality due to over- or underexposed regions that induce errors in the measured spectrum and, consequently, in classification.

Reported underwater HSI systems have mostly employed near-Lambertian light sources, particularly halogen [47] or LED [44] lamps. With our novel design, the angular distribution of the light source could be narrowed while still preserving uniform illumination. The peak irradiance across the FOV of the camera at the target surface was estimated to be of the order of 120 W/m${}^{2}$. For reference, simulations demonstrated that the intensity would have decreased six-fold in case the aspheric condenser was not used. This demonstrates the relevance of having focused or collimated light sources to increase the backscattering signal levels and thereby allow for faster acquisitions.

Compared with push broom systems, the main advantage of our staring-type imager is the possibility to independently adjust the exposure time for each wavelength channel to maximize SNR. Since a good SNR is critical for high spectral reliability [44], underexposed regions must be avoided to ensure that the hypercube has a higher dynamic range. On the other extreme, overexposure disrupts linear transformations. In push broom systems, all wavelength channels are acquired simultaneously with a common integration time. This is particularly problematic if the LED spectrum is not tailored to ensure a nearly flat spectral response of the full system since, for instance, absorption in water is stronger towards the red. This is the case in [47], where wavelength bands above 691 nm and below 470 nm are rejected due to low SNR, as well as in [26] for λ<450 nm. The rejection of these bands potentially eliminates vital spectral fingerprints from the analysis (in our case this would reject the chl-a absorption peak at 450 nm and autofluorescence at >700 nm). In our system, the exposure times near the limit bands were simply increased to increase the SNR ($SNR\propto t_{exp}$). Notwithstanding, this led to longer acquisition times. The sum of the exposure times for the full hypercube acquisition herein was around 6.6 seconds; if the bands <470 nm and >691 nm were ignored, this time would have decreased to around 1.9 seconds.

The biggest comparative downside of our system is the considerably worse spectral resolution. The FWHM of the employed LCTF was between 6.8 and 14.4 nm, while underwater push broom systems can achieve maximum spectral resolutions of 0.5 nm [22]. This means that, under our system, narrow spectral features appear smeared out. Nonetheless, spectral features from solid and biological targets are, in general, relatively broad-banded, in contrast to sharp well-defined features observed from gases [61]. Accordingly, our results indicate that fine spectral resolution might not be needed to discriminate relevant biofouling species at the group level according to the spectrum. However, finer spectral resolution might be needed for more detailed taxonomic analysis and identification.

### 4.2. Spectral Library of Biofouling Species

A library of spectral signatures was constructed for representative biofouling species collected at the CMTC. The raw spectral data were calibrated to convert digital counts into reflectance values, and thus correct for wavelength-dependent system and propagation medium parameters. Characteristic imprints arising from absorption and autofluorescence of chl-a pigments were observed in the spectral reflectance signatures of all algae species and played an important role in the classification step.

During this stage, and to make the classification model more robust and accurate, it is important to account for intra-species variability in the optical signatures due to differences, among others, in density and health status. For this reason, a large amount of spectra needs to be collected which constitutes a very time-consuming task. Moreover, an accurate calibration procedure is also fundamental since trustworthy and consistent signatures are required for rigorous classifications. Due to the non-uniformity of the illumination and LCTF transmission over the CA, a pixel-wise calibration was applied to simultaneously perform a flat-field correction and reflectance conversion, thus making all the pixels directly comparable.

A standard with quasi-100% reflectance is often used as the reference for the calibration (e.g., spectralon or polyethylene [47]). However, it has been argued that this is not strictly required [36,45]. Considering the low reflectance of most biofouling organisms and that the standard needs to be imaged under the same exposures as the hypercubes, we opted to use 10% reflectance standard to maximize the dynamic range. Equation (2) generalizes the transformation to reflectance for an arbitrary reflectance standard. The obtained red algae signatures (Figure 8) are comparable with a past study [26], both quantitatively (absolute reflectance) and qualitatively, despite the differences in reflectance standards used for calibration (10% vs. 99%). This suggests that our calibration procedure was accurate.

### 4.3. Supervised Classification of Submerged Biofouled Panels

The overall objective of this study was to develop a technique to detect, classify, and quantify biofouling on coated surfaces and, thus, generate an objective and repeatable method to evaluate the performances of FCCs. Using the spectral library as dataset to train a WNN model allowed us to map the distribution of the different classes on new panels (only requiring a measured and calibrated hypercube). The accuracy of the classifier was tested on a spatially disjoint model target with known ground truth to evaluate its performance. The overall classification accuracy of the WNN classifier was estimated to be around 95%. The recall rate was superior to 90% for all classes, with a minimum of 90.8% for red algae and a maximum of 99.2% for the panel class. The precision was above 98% for all classes besides for brown algae which registered a rate of 81.0%. This indicates that, when grouped according to color categories, the macroalgae can be correctly classified to a great extent with our HSI system. The classification capabilities were also tested in real fouled panels and a comparison of the segmented images with direct visual inspection of color images revealed a substantial agreement. Whatsoever, the accuracy was not quantified as it would require correct identification of the species on the panel for ground truth, which was difficult to determine with the naked eye due to the intertwined growth.

Concurrently, when we considered a separately-trained neural network with more detailed description of the classes taxonomy-wise, the overall accuracy on the model target dropped to 81.5%, driven by substantial confusion among algae species within the same color group. Even within the same taxon, the algae color can change, so the collected spectra are not unequivocally linked to a particular species. This indicates that mapping independently each algae species with high-accuracy and without extra information besides the spectrum might prove a difficult task. Furthermore, in the maritime context, the number of species that can be observed is very large and taxonomy is highly complex, so a species-based approach is expected to scale poorly [15].

Classification on real panels was in line with qualitative observations under visual inspection. There were, however, some ambiguities at the boundary regions of the panel, even to the naked eye. Because the settlement of biofouling is a gradual and dynamic process, the build-up on the panel occurs in different stages [62]. Hence, throughout the biofouling growth process and until the organism reaches a stage in which it mostly dominates the pixel spectrum, one can expect to have mixed pixels signatures that are a combination of panel and organism spectra (that also change according to its status). Since the training dataset only includes sample points for both clean panels and fully grown macroalgae, these mixed pixels introduce confusion in the WNN classifier (see for instance the region marked with a red circle in the panel coverage image). To improve the accuracy of the model and circumvent confusion, the dataset needs to be further expanded to include algae in different stages of development.

When compared with RGB camera-based systems [15,16,19], HSI extends the spectral information to tens of wavelength channels. This way, characteristic biofouling fingerprints that would otherwise be missed by color cameras were resolved. This was crucial to increase the classification specificity, allowing us to go beyond the semi-quantitative classification in a three-level holistic scale proposed in [15] for vessels. Among the most prominent spectral features herein, the absorption and autofluorescence signatures of chl-a revealed central to discerning algae from non-algae. In the context of evaluating the performances of FCCs, having more granular detail about the coverage percentage of both biofouling as a whole and specific groups is valuable to support the development stages and the direct comparison between different coatings. Thus, our system permitted the quantification of coverage of the clean panel, three groups of algae (green, red, brown), and two animals (mussels and barnacles). These coverage percentages can stand alone as evaluations of performances or can be combined with existing evaluation standards for the performances of FCCs, such as the ECHA [13]. Using [19] as a reference, the accuracy of an underwater RGB camera combined with an SVM model was, overall, 58%, with the TPR peaking at 75% for the tunicates. Our HSI system combined with the WNN model accomplished an overall accuracy of 95%, with a minimum TPR of above 90% for red algae. Although the classes considered were distinct, the discrepancies were regarded as a good indicator of the advantages of HSI compared to RGB cameras.

The advantages of the developed staring-type HSI come at the expense of some drawbacks. Primarily, the acquisition of multiple spectral bands inevitably means longer acquisition times (in this case, 63 images vs a single snap-shot required with a color camera). To soften integration times and fasten the throughput, we combined an EMCCD with a high-power LED lamp to boost photon-budget. Having acquired a full hypercube, WNN classification occurs virtually instantaneously, taking sub-second times to assign a class to every pixel in the frame. Therefore, HSI is still compatible with agile measurements required to unlock large area analysis. The LCTF addition also means a bigger footprint and a more expensive system.

All the processing, analysis, and classification were purely based on spectral features. No spatial contextualization was considered. Nevertheless, one can take advantage of the spatially-rich information retained by 2D imaging sensors in HSI and incorporate it into the model to support classification. Since the shape is usually an important identifying element, a spectral-spatial classifier with enhanced specificity and accuracy can be built to simultaneously consider morphological and spectral information. The implementation of these additional features into a classifier requires more advanced models, often deep convolutional neural networks [52,63], which try to mimic human visual recognition. A study ought to be carried out to evaluate the most suited algorithm, as it has been demonstrated that it has the potential to affect the classification outcome [26], followed by an optimization procedure to fine-tune the model parameters. This was left out of the scope of this pilot study.

All the results were obtained from in vivo measurements in a controlled environment as this was a pilot to study the feasibility of spectral imaging applied to the evaluation of marine coatings. In the future, the sensor ought to be submerged and tested in situ to include the effects of increased water volume and, therefore, increased absorption, scattering, and fluorescence from dissolved organic matter and suspended chl-a, which will certainly degrade the SNR and contrast [26]. For underwater deployment, a hermetically-sealed housing is required with a dome port to correct the magnification due air-glass window-water refractive index mismatch. The system can be fixed to an underwater platform observing the panels at a constant distance and within a consistent FOV.

Overall, the application of HSI for biofouling assessment is a novel contribution to the automated description and evaluation of the performances of FCCs. The proposed technique combined with a novel light source with high uniformity and power can swiftly and reliably produce maps of the biofouling coverage per class on panels that can afterward be analyzed to produce objective assessments. This pilot study demonstrated that HSI is a superior alternative to current imaged-based methodologies, both specificity and accuracy-wise, due to the extended spectral information. Moreover, since the procedure is automated, it can accomplish objective and repeatable evaluations when compared to traditional methods. Hence, HSI has the potential to replace manual or photointerpretation techniques that are time-consuming and have, up until this point, depended on specialized human examiners with their inherent subjectivity [14,15,16]. As a result, this powerful tool has the potential to provide valuable information that will facilitate the task performance evaluations of FCCs, both on spatial and temporal scales. Moreover, HSI is a remote sensing technology and therefore non-destructive by nature, so in situ and in vivo measurements are possible without interfering with the fouling dynamics. Besides improving the comparability between FCCs, the categorized output will also inform about preferential growth of biofouling classes. This system can therefore provide information on the fouling dynamic that will be crucial to support the R&D of new environmentally friendly FCCs. Further developments will permit higher throughput and more comprehensive classification taxonomy-wise that will further deepen the level of detail of the assessments.

## Data Availability

The data presented in this study are openly available on FigShare at https://doi.org/10.6084/m9.figshare.20607867.

## Supplementary Materials

The following supporting information can be downloaded at https://www.mdpi.com/article/10.3390/s22187074/s1. The supplementary document includes information about the samples distribution among classes, the results obtained for the fine grouping of classes, and images of segmentation of additional real panels.

## Abbreviations

The following abbreviations are used in this manuscript:

ASTM	American Society for Testing and Materials
CA	clear aperture
CMTC	CoaST Maritime Test Centre
CWL	center wavelength
ECHA	European Chemicals Agency
EMCCD	electron multiplying charge-coupled device
FCC	fouling control coating
FDR	false discovery rate
FNR	false negative rate
FOV	field of view
FWHM	full-width at half-maximum
HSI	hyperspectral imaging
LCTF	liquid crystal tunable filter
LED	light-emitting diode
NA	numerical aperture
NIS	non-indigenous species
NSTM	naval ships’ technical manual
PC	principal component
PCA	principal component analysis
PPV	positive predictive value
ROI	region of interest
SNR	signal-to-noise ratio
SVM	support vector machine
TPR	true positive rate
WNN	wide neural network
