# Supplementary material for: A Tunable Hyperspectral Imager for Detection and Quantification of Marine Biofouling on Coated Surfaces
Source: Sensors (Basel). 2022 Sep 19;22(18):7074. doi: 10.3390/s22187074 (PMC9505677; doi:10.3390/s22187074)
Supplement: Supplementary file 1 [file sensors-22-07074-s001.zip › sensors-1857392-supplementary.pdf]

Article

Supplementary materials: A Tunable Hyperspectral Imager for Detection and Quantification of Marine Biofouling on Coated Surfaces

Joaquim Santos [1,‡], Morten Lysdahlgaard Pedersen [2,3,4,‡], Burak Ulusoy [2,3,4], Claus Erik Weinell[2], Henrik Chresten Pedersen[1], Paul Michael Petersen[1], Kim Dam-Johansen[2], and Christian Pedersen [1,*]

1 Department of Electrical and Photonics Engineering (DTU Electro), Technical University of Denmark, 4000 Roskilde, Denmark

2 CoaST, Department of Chemical and Biochemical Engineering (DTU Chemical Engineering), Technical University of Denmark, 2800 Kongens Lyngby, Denmark

3 Sino-Danish Center for Education and Research, 100093 Beijing , China

4 Sino-Danish College, University of Chinese Academy of Sciences, 100049 Beijing, China

‡ These authors contributed equally to this work, as first authors.

Supplementary Materials:

The following document contains the supplemental material for the article *A Tunable Hyperspectral Imager for Detection and Quantification of Marine Biofouling on Coated Surfaces*. The supplementary material includes a table with the datapoints distribution among the classes (Table S1), the new spectral library, confusion matrix, and coverage per species after classification of the fine Wide Neural Network (WNN) in Figure S1, S2, and S3, respectively. The fine WNN was trained using the same algorithm and parameters. However, a differently annotated training and test set (sampled from the same ROIs) were used this time with the species labels; two green algae - *Ulva sp.*, *Zostera sp.*; one red algae - *Ceramium sp.*; six brown algae - *Petalonia sp.*, *Scytosiphon sp.*, *Desmarestia sp.* (2 different subspecies), *Dictyosiphon sp.*, and *Chorda sp.*. The collected animals were *Balanus sp.* (barnacles) and *Mytillus sp.* (mussels). Furthermore, an additional real fouled panel was evaluated at three fouling states; fully fouled, medium fouled, and low fouled. The segmentation results for each of the three states of fouling are shown in Figure S4. In Figure S5, the coverage per species after classification with the in paper presented model are shown, alongside with a RGB image of the panel.

Table S1. Distribution of the training and test sets among the different classes.

Class (Coarse Grouping)	Species (Fine Grouping)	Number of datapoints (Training set)	Number of datapoints (Test set)
Green Algae	*Ulva sp.*	7482	7581
	Zostera sp.	9897	2932
Brown Algae	*Petalonia sp.*	1678	1804
	Scytosiphon sp.	5894	2613
	Desmarestia sp.	1901	836
	Desmarestia sp. (2)	13069	-
	Dictyosiphon sp.	86943	-
	Chorda sp.	11280	1164
Red Algae	*Ceramium sp.*	12236	11758
Mussel	*Mytilus sp.*	26641	-
Barnacle	*Balanus sp.*	632	-
Blue Panel	=	52500	-
Blue FCC Panel	=	42500	-
White Panel	=	19609	-
Red Panel	=	45000	-
Grey Panel	=	56395	12814
Grey Panel 2	=	45000	-
	**Total**	438657	41502

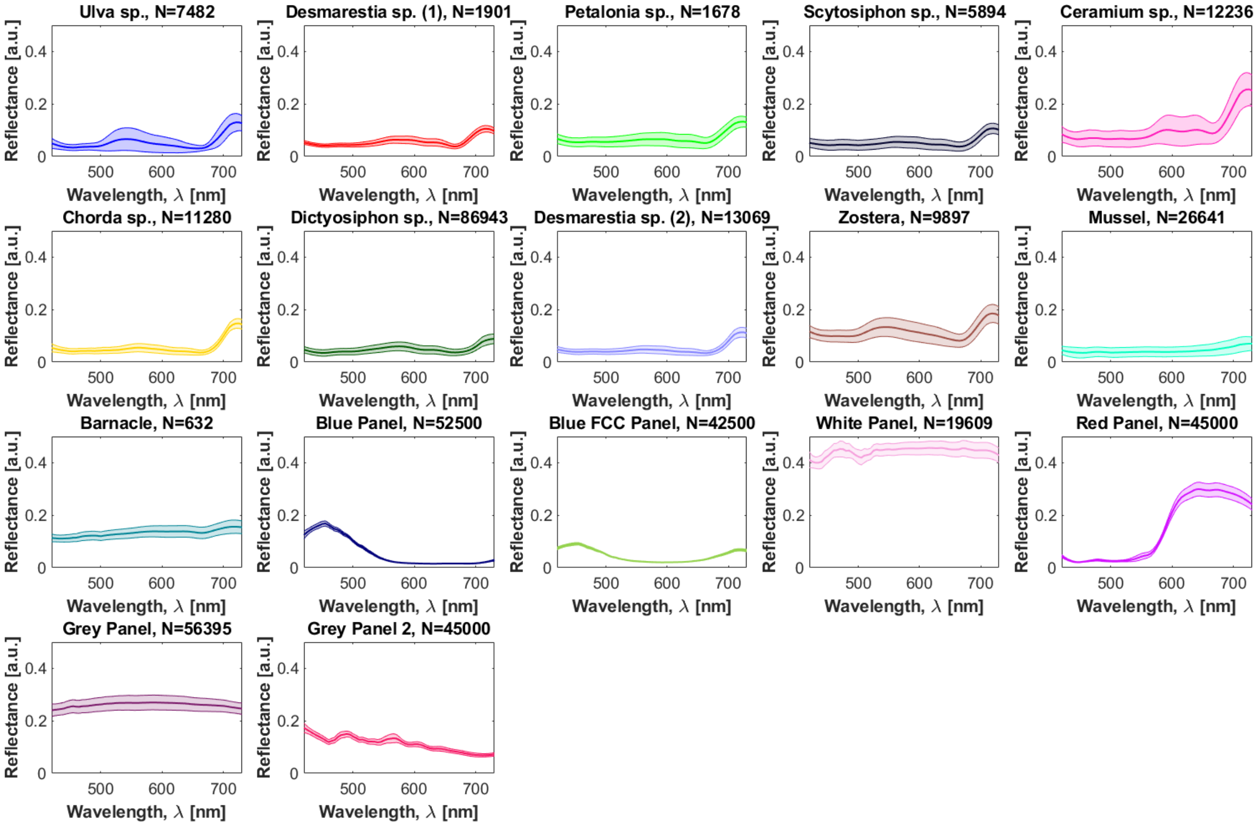

Figure S1. Mean signatures from the spectral library used as training set for the fine Wide Neural Network (WNN) model. The shaded regions represent the standard deviation.

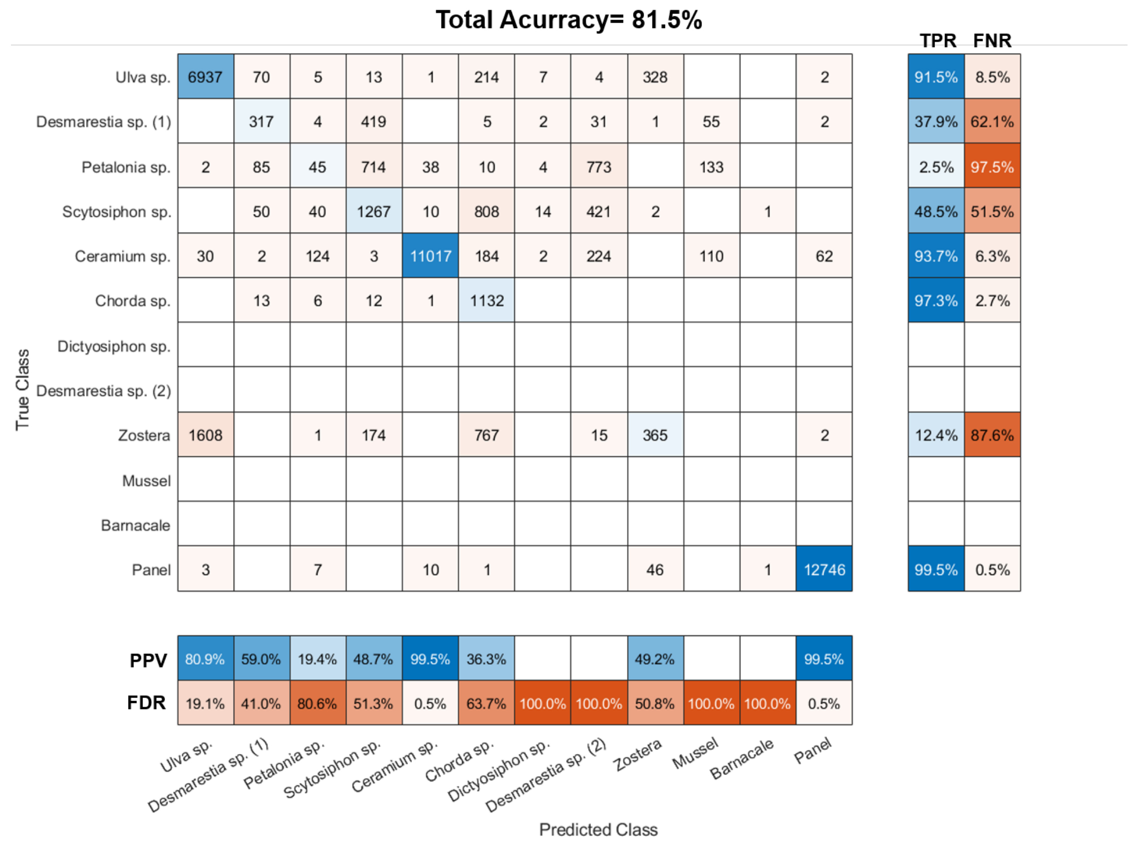

Figure S2. Confusion Matrix for the fine WNN classification of the model target with ground truth in rows and predictions in columns. The True Positive Rate (TPR) and False Negative Rate (FNR) for each class are presented on the side; the Positive Predictive Value (PPV) and False Discovery Rate (FDR) are presented below. No mussels or barnacles were present in the model target.

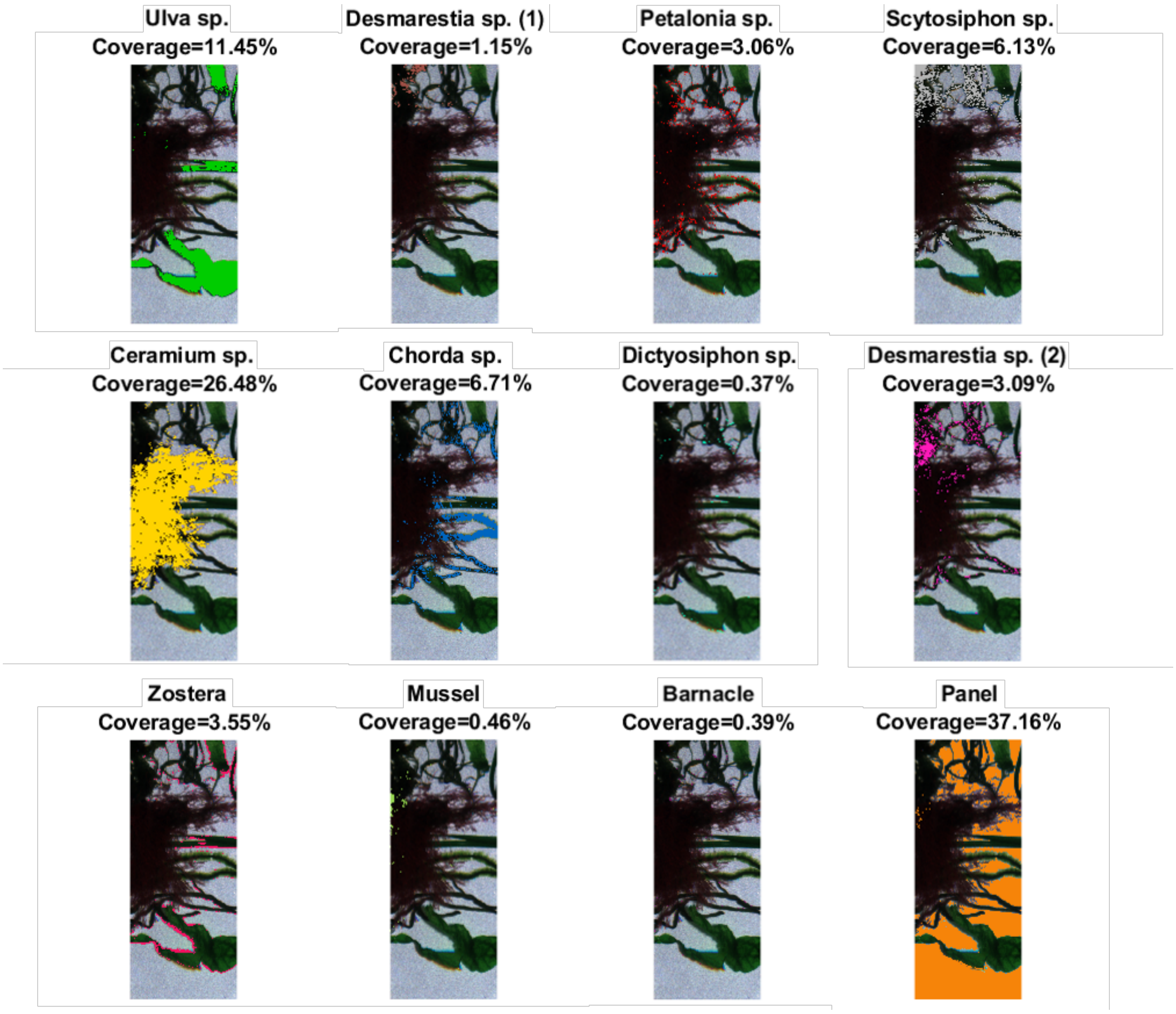

Figure S3. Maps of each category obtained by the fine WNN classification on the model target, including coverage percentages. The underlying image was obtained by selecting three RGB bands from the hypercube. The overlay indicates the pixels classified as the corresponding class.

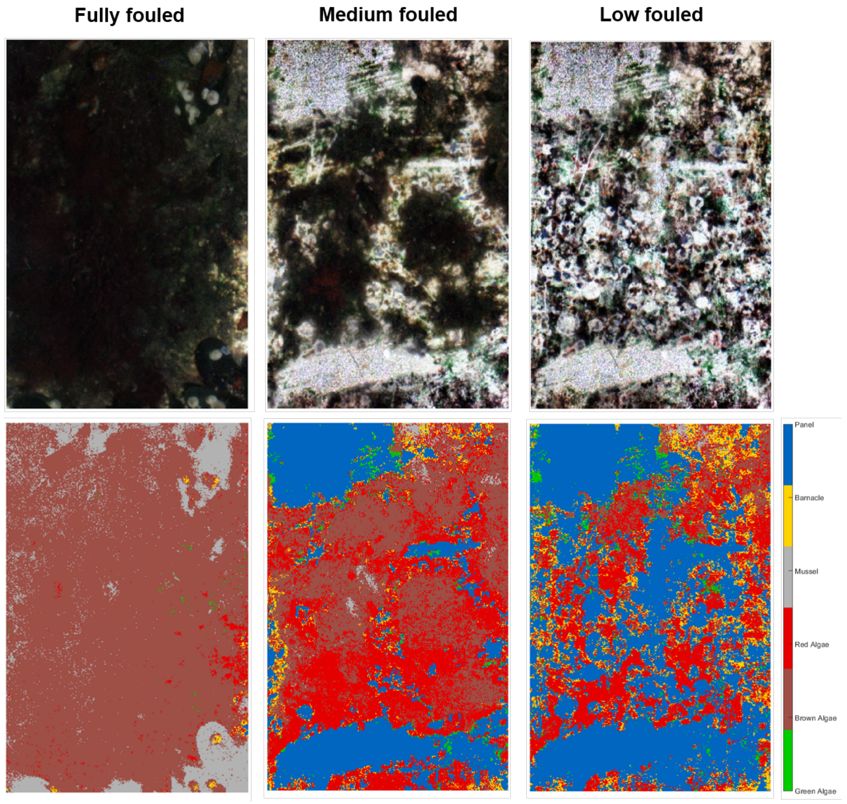

Figure S4. A coated panel after more than one year exposure at CMTC is classified using the WNN. The classification is done in multiple steps as the panel was scrapped for fouling to represent three states of fouling, fully, medium, and low fouled.

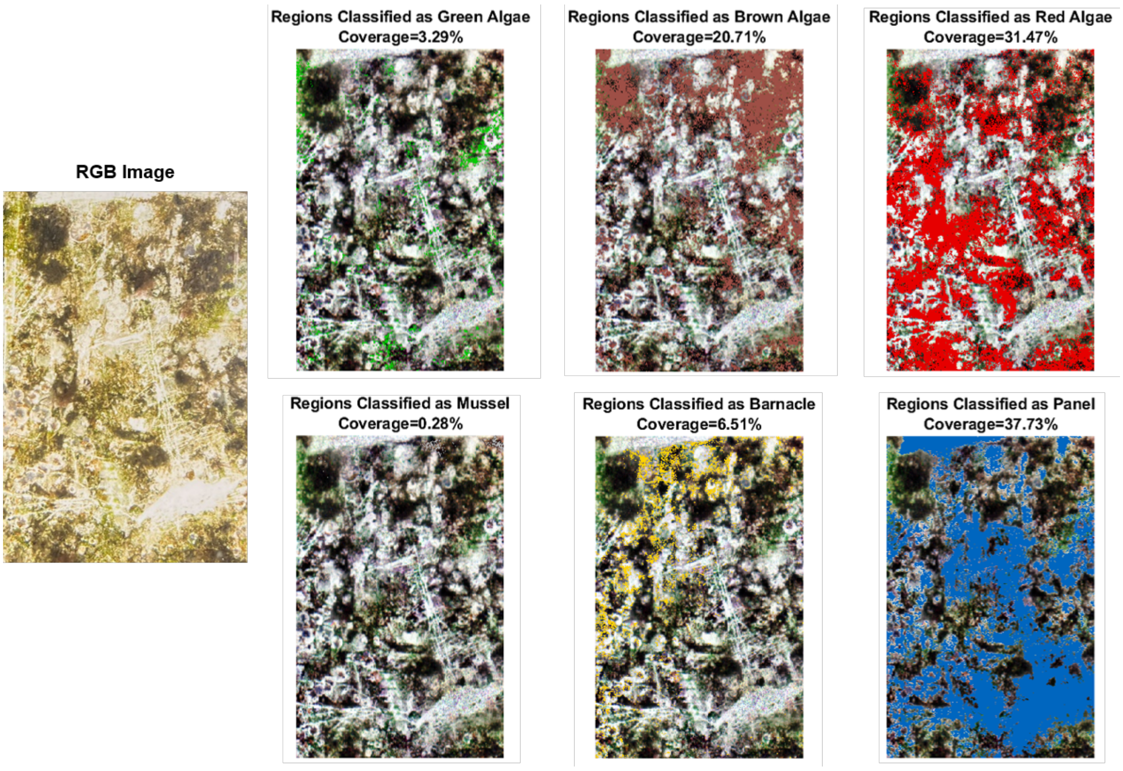

Figure S5. Classification and coverage percentage of biofouling for the low fouled panel obtained from using the WNN. On the leftmost side, the RGB image of the panel taken with a commercial camera is shown.
